# Local and Systemic Micro-Rheological Changes during Intestinal Anastomosis Operation: A Metabolic Dependence in an Experimental Model

**DOI:** 10.3390/metabo14080458

**Published:** 2024-08-18

**Authors:** Adam Varga, Adam Attila Matrai, Barbara Bedocs-Barath, Laszlo Adam Fazekas, Felipe Salignac Brasil, Aashna Mehta, Erzsebet Vanyolos, Adam Deak, Tamas Lesznyak, Katalin Peto, Norbert Nemeth

**Affiliations:** Department of Operative Techniques and Surgical Research, Faculty of Medicine, University of Debrecen, Moricz Zsigmond u. 22, H-4032 Debrecen, Hungary; varga.adam@med.unideb.hu (A.V.); matrai.adam@med.unideb.hu (A.A.M.); barath.barbara@med.unideb.hu (B.B.-B.); fazekas.laszlo@med.unideb.hu (L.A.F.); salignac.brasil@med.unideb.hu (F.S.B.); mehta@mailbox.unideb.hu (A.M.); vanyolos@med.unideb.hu (E.V.); deak.adam@med.unideb.hu (A.D.); lesznyak.tamas@med.unideb.hu (T.L.); kpeto@med.unideb.hu (K.P.)

**Keywords:** hemorheology, microcirculation, intestinal anastomosis, red blood cell deformability, osmotic gradient ektacytometry, red blood cell aggregation

## Abstract

Hemorheological factors may show arterio-venous differences. Alterations in acid-base and metabolic parameters may also influence these factors. However, little is known about changes in micro-rheological parameters during abdominal surgery, influencing splanchnic circulation. In anesthetized pigs, the external jugular vein, femoral artery and vein were cannulated unilaterally, and paramedian laparotomy was performed. In the anastomosis group, after resecting a bowel segment, end-to-end jejuno-jejunostomy was completed. Blood samples (from cannulas and by puncturing the portal vein) were taken before and after the intervention. Hematological, acid-base and blood gas parameters, metabolites, red blood cell (RBC) deformability and aggregation were determined. The highest hematocrit was found in portal blood, increasing further by the end of operation. A significant pH decrease was seen, and portal blood showed the highest lactate and creatinine concentration. The highest RBC aggregation values were found in arterial, the lowest in renal venous blood. The RBC aggregation increased with higher lactate concentration and lower pH. Osmotic gradient deformability declined, with the lowest values in portal and renal venous samples. In conclusion, micro-rheological parameters showed arterio-venous and porto-renal venous differences, influenced by oxygenation level, pH and lactate concentration. The intestinal anastomosis operation caused an immediate micro-rheological deterioration with portal venous dominancy in this experiment.

## 1. Introduction

Hemorheology describes the macro- and microscopic-dimensional flow of the blood’s cellular and plasmatic components and the vascular wall’s rheology in contact with the blood [1]. Blood exhibits non-Newtonian fluid behavior, meaning that its viscosity varies with the velocity gradient and can be represented by a characteristic Casson-type curve. The primary factors influencing blood viscosity include plasma viscosity, hematocrit, red blood cell deformability and aggregation [2,3]. Deterioration of these factors can lead to impaired tissue microcirculation and blood supply [4,5]. Certain external factors can cause a lack of blood supply to the body, resulting in hypoxia, I/R injuries, acidosis, and metabolic disturbances [6,7,8]. Triggers of changes such as tissue damage, inflammation, mechanical trauma, and free radicals induce metabolic and structural changes in red blood cells, leading to the deterioration of micro-rheological parameters (erythrocyte deformability and aggregation). As part of the acute phase reactions, the increase in hemoconcentration and fibrinogen concentrations directly increases blood and plasma viscosity and enhances aggregation. The ensuing microcirculatory disturbances, endothelial dysfunction, and reduction in blood fluidity induce further perfusion disturbances. It is already known that certain surgical interventions can also induce acute phase reactions and serve as a natural defense against tissue injury, promoting repair and healing [9,10,11]. Moreover, major surgeries, including cardiac interventions, organ transplants, and extensive abdominal procedures, frequently trigger significant inflammatory responses [12,13]. Surgeries involving the heart, blood vessels, and organ removal can trigger acute phase reactions, releasing inflammatory mediators crucial for immune response and healing. While symptoms like fever, pain, swelling, and increased heart rate are common, they are temporary and considered normal, facilitating tissue regeneration after surgical trauma [14]. Additionally, acute-phase proteins contribute to clotting, tissue repair, and immune modulation [15,16].

We have chosen one of the important steps in Roux-en-Y bypass (RYGB) surgery, the jejuno-jejunostomy, as the subject of our research [17]. It holds significance in numerous surgical interventions, such as bypass surgeries, and can be conducted via laparoscopic techniques [18]. Despite its utility, jejuno-jejunostomy carries potential complications, necessitating careful management through suitable medical and surgical approaches. Possible complications encompass infections, bleeding, strictures, ulcers, intestinal obstruction, thromboembolism, and malnutrition [19]. From a surgical point of view, the most important step in a well-performed jejuno-jejunostomy is to determine the correct position of the bowel segment to be resected. A small bowel resection is a surgery to remove part of the small intestine, often performed to treat obstructions, diseases, cancer, Crohn’s disease, large polyps, and bowel injuries [20]. Reasons for needing a small bowel resection include intestinal blockages, bleeding, infection, inflammatory bowel diseases like Crohn’s disease, regional ileitis, or regional enteritis, noncancerous tumors or precancerous polyps, small intestine cancer, injuries to the small intestine and Meckel’s diverticulum [20].

Previous studies have revealed that hemorheological parameters may also show arterio-venous differences and portal venous blood samples may also differ micro-rheologically [21]. However, the data are still debated. In a prior rat study, we observed higher aggregation index values and lower elongation index values in arterial blood from the abdominal aorta compared to venous blood from the caudal caval vein [22]. This arterial-venous hemorheological difference has been corroborated by other researchers [23], although some studies did not find significant differences and noted higher aggregation index values in venous blood [24]. These experimental models indicate that porto-caval hemorheological differences should be considered when planning and interpreting experiments. To make this phenomenon more comprehensible, osmotic gradient ektacytometry (osmoscan) parameters may help to understand these differences. The osmoscan facilitates the analysis of red blood cell (RBC) deformability across osmotic gradients. Monitoring changes in RBC deformability alongside alterations in osmolality offers insights into RBC behavior across diverse osmotic environments. 

We hypothesized that the micro-rheological characteristics of blood deteriorate during intestinal surgery, and the extent of these alterations varies across distinct blood sampling locations. Based on this hypothesis, we aimed to perform micro- and macro-rheological analysis of systemic arterial, venous, portal, and renal blood samples, and to study the changes in blood gas and metabolite parameters before and after small bowel anastomosis in a porcine model.

## 2. Materials and Methods

### 2.1. Experimental Animals

Sixteen female Hungahib-39 (Agrargazdasag Ltd., Debrecen, Hungary) hybrid pigs were used in our study (ethical permission registration No.: 24/2016/UDCAW, 16/2018/UDCAW). The experiments were performed by The Hungarian Animal Protection Act (Law XXVIII/1998). The average age of the animals was 7–8 weeks and 18.93 ± 1.88 kg was the average body weight. Before surgery, the animals underwent a standard acclimatization period during which they were deprived of food for 16 h. This study was performed during the “Advanced Surgical Operative Techniques” compulsory elective course in the 2022 autumn semester.

### 2.2. Surgical Protocol

The anesthesia protocol was the following: for pre-medication, i.m. 1–2 mg/kg of azaperone (Stresnil, Elanco GmbH, Cuxhaven, Germany); for induction of anesthesia, i.m. 2 mg/kg of xylazine (CP-xylazine hydrochloride, 2%) and 20 mg/kg of ketamine (CP-ketamine hydrochloride 10%); for maintenance of permanent anesthesia, i.v. 1 mg/kg of xylazine and 10 mg/kg of ketamine, supplemented with i.v. 2 mg/kg of diazepam (Diazepeks 5 mg/mL, AS Grindeks, Riga, Latvia). After anesthesia, a tracheal tube (ID 5.5; Eickemeyer, Tuttlingen, Germany) was inserted for assisted ventilation. Before the surgery the animals were divided into two equal groups randomly: an anastomosis group and a sham-operated control group. Afterward, the left external jugular vein was gently prepared, exposed and cannulated directly for i.v. 10 mL/kg/h fluid replacement (amount: 452.4 ± 45.9 mL, “Baxter” Sodium Chloride 0.9%, pH = 4.5–7.0, osmolarity: 308 mOsm/l, Baxter Hungary Ltd.). The left femoral artery and the right femoral vein were used as systemic blood sampling sites after cannulation (Certofix Duo S730 B. Braun SE, Hessen, Germany). The paramedian laparotomy started with preoperative preparation. The pig’s abdomen was meticulously disinfected and isolated to provide a sterile surgical field followed by an incision through the skin. To facilitate the exploration, isolation lines were established. Once hemostasis was achieved, we proceeded to make an incision in the peritoneum. In the sham-operated control group, this was the last step of the surgical intervention and the closure of the abdomen.

In the anastomosis group, we performed an end-to-end jejuno-jejunostomy. After intestinal clamps, we resected a bowel segment (the average length of the resected bowel segments was 12 ± 0.65 cm). To perform the anastomosis, we used a one-layer suture line with Mikulicz-stitches (suture material: VITREX Monolac Monofil Violet, USP 4/0, Vitrex Medical A/S, Herlev, Denmark). The average time of the experimental operation was 143 ± 12 min (from starting anesthesia until the last blood sampling immediately after completing the anastomosis). The average time of completing the small bowel anastomosis was 38 ± 7 min. 

### 2.3. Blood Sampling

During the experiment, 3.5 mL of blood was taken at two time points and from a total of four different locations. Before abdominal surgery, blood was drawn from the femoral artery and the femoral vein using pre-inserted cannulas. The blood was collected into standard Vacutainer tubes (BD Vacutainer^®^ tubes, 1.8 mg/mL K3-EDTA; Becton, Dickinson and Company, Franklin Lakes, NJ, USA). The renal vein and portal vein were also sampled but that was carried out by direct puncture with a 23 G needle and 5 mL syringe after the median laparotomy. Blood samplings were completed after the end-to-end jejuno-jejunostomy starting with the systemic cannulas, then the portal and the renal veins by direct puncture. In the sham-operated control group, we kept the same protocol. 

### 2.4. Hematological Measurements

A Sysmex K4500 microcell counter device (TOA Medical Electronics Corp., Ltd., Kobe, Japan) was used to determine RBC count [10^12^/L], white blood cell count (WBC [10^9^/L]), hemoglobin concentration (Hgb (g/dL)), and platelet count (Plt [10^9^/L]). Calculated values by the automate were hematocrit (Hct [%]), mean corpuscular volume (MCV [fL]), mean corpuscular hemoglobin (MCH [pg]) and mean corpuscular hemoglobin concentration (MCHC [g/L]).

### 2.5. Hemorheological Measurements

Red blood cell deformability was tested using a LoRRca Maxsis Osmoscan ektacytometer (RR Mechatronics International B.V., Zwaag, The Netherlands) [25]. In this ektacytometry method, RBCs were subjected to shear stress, and their elongation was determined by laser diffraction techniques. The so-called elongation index (EI) was described by the function of shear stress (SS [Pa]). For the conventional deformability test, 10 μL of whole blood was gently mixed with 2 mL of polyvinyl-pyrrolidone (PVP)–PBS solution (PVP: 360 kDa, Sigma-Aldrich Co., St. Louis, MO, USA; PVP-PBS solution viscosity = 28–32 mPas, osmolality = 290–310 mOsmol/kg, pH= 7.2–7.5). All measurements were carried out at 37 °C. From the EI–SS curves, comparative data were used, such as EI values at 3 Pa, and by parameterization of the entire EI–SS curve, maximal elongation index (EI_max_) and shear stress at half EI_max_ (SS_1/2_, [Pa]), which were calculated based on the Lineweaver–Burk equation [26].

Osmotic gradient deformability (osmoscan) measurements were carried out using 5 mL of isotonic PVP-PBS that was mixed with 250 μL of blood. During the measurements, the determination of EI was performed at constant shear stress (30 Pa), while the osmolality of the suspension changed as the device mixes low-osmolar (0 mOsm/kg) and high-osmolar (500 mOsm/kg) PVP solutions with the whole-blood sample. The blood sample was aspirated into this PVP solution with a gradually increasing osmolality, while the elongation index was continuously registered. The result was a characteristic EI–osmolality (O) curve, with several notable points. EI_min_ represents the minimal elongation index in the low-osmolar environment. The associated osmolality value, O_min_ (osmolality at EI_min_), roughly corresponds to an osmolality value where 50% of the RBCs hemolyse in the osmotic fragility test. EI_max_ here means the maximal elongation index in the function of osmolality (note: it is not the same as EI_max_ that was calculated by the Lineweaver–Burk equation). Osmolality at EI_max_ (O (EI_max_) is the value where RBCs deform optimally. EI_hyper_ (half of the maximal elongation index in the high-osmolar environment) and O_hyper_ show the point in the hyperosmolar region where the RBCs are half of their maximal elongation. Another parameter is the Area, which is calculated from the area under the individual EI–O curves.

Besides the standard comparative parameters of the osmoscan curves, further parameters were calculated: ΔEI (absolute difference in maximal and minimal EI values), ΔO (absolute difference in osmolality values at maximal and minimal EI), and ratio values: EI_max_/EI_min_ (rEI), O (EI_max_)/O_min_ (rO), ΔEI/ΔO, and rEI/rO [27].

A Myrenne MA-1 erythrocyte aggregometer (Myrenne GmbH, Roetgen, Germany) was used to determine the RBC aggregation [28]. The test requires approximately 20 µL of blood. After disaggregation by a controlled shearing system (shear rate: 600 s^−1^), the light transmission was tested for 5 or 10 s at stasis (M values, shear rate: 0 s^−1^) or at a low shear (M1 values, shear rate: 3 s^−1^). The higher index values represent enhanced red blood cell aggregation.

### 2.6. Blood Gas, Acid-Base Parameters, Metabolites and Electrolytes

To analyze the blood gas and acid-base parameters, we used the EPOC Blood Analysis System (Siemens Healthineers AG, Erlangen, Germany). The following parameters were measured: pO_2_ [mmHg], pCO_2_ [mmHg], pH, cHCO_3_^−^ [mmol/L], Na^+^ [mmol/L], K^+^ [mmol/L], Ca^2+^ [mmol/L], Cl^−^ [mmol/L], glucose [mmol/L], lactate [mmol/L] and creatinine [µmol/L] concentrations.

### 2.7. Statistical Analysis

The Mead’s resource equation method was employed to determine the required sample size for the experiment. Statistical analyses were conducted using SigmaStat Software 3.1.1.0. (Systat Software Inc., San Jose, CA, USA). Descriptive statistics are presented as means ± standard deviation (S.D.). Based on the results of the normality test, inter-group differences were assessed using either the *t*-test or the Mann–Whitney rank-sum test, while one-way ANOVA or Kruskal–Wallis’s test were used for intra-group comparison. A *p*-value lower than 0.05 was considered statistically significant.

## 3. Results

### 3.1. Hematological Parameters

The white blood cell count significantly decreased in the anastomosis group in every blood sampling site compared to the base measurements (portal and renal venous blood: *p* < 0.001, artery: *p* = 0.023, vein: *p* = 0.01). The red blood cell count increased after the bowel surgery in all samples (*p* < 0.001). In the case of hemoglobin concentration, we observed that after the bowel surgery, the systemic arterial, venous, and portal venous samples showed a significant increase compared to the base and control values (*p* = 0.002 vs. before operation, *p* = 0.004 vs. control femoral artery, *p* < 0.001 vs. control femoral vein), while the hematocrit increased mainly in the portal and renal venous blood samples (*p* < 0.001 vs. before operation, *p* = 0.01 vs. control portal vein, *p* < 0.001 vs. renal vein). The mean corpuscular volume increased in every sample after operation in the anastomosis group (*p* < 0.001 vs. before operation) and this phenomenon was also present when compared with the control group (*p* = 0.02 vs. control femoral artery, *p* < 0.001 vs. control femoral vein, *p* = 0.023 vs. control portal vein, *p* < 0.001 vs. renal vein). There was no significant change in the MCH and MCHC parameters. In contrast, platelet count significantly decreased in both the control and the operated groups, and further changes were observed after the intervention (*p* < 0.001 vs. control femoral vein, *p* = 0.015 vs. control portal vein, *p* < 0.001 vs. renal vein) (Table 1).

### 3.2. Red Blood Cell Deformability

**Figure 1:**
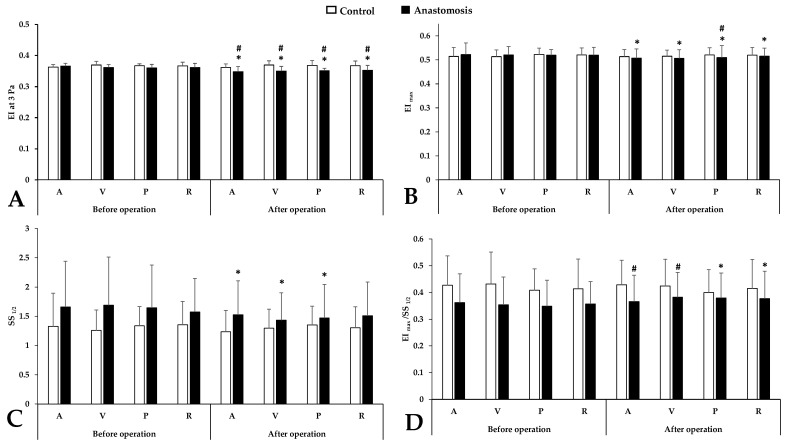
Alterations in conventional red blood cell deformability parameters in systemic arterial (A), venous (V), portal venous (P) and renal venous (R) blood taken before and after operation in the control and anastomosis groups. (**A**): EI at 3 Pa; (**B**): maximal elongation index (EI_max_); (**C**): shear stress at the half of EI_max_ (SS_1/2_ [Pa]); (**D**): ratio of EI_max_ and SS_1/2_ Means ± S.D.; * *p* < 0.05 vs. before operation (base), # *p* < 0.05 vs. control.

EI at 3 Pa results (Figure 1A) expressed major significant differences between the control and anastomosis groups after the intervention (*p* < 0.001 vs. control femoral artery, *p* < 0.001 vs. control femoral vein, *p* < 0.001 vs. control portal vein, *p* < 0.001 vs. control renal vein) and all sampling points in the anastomosis group showed a significant decrease compared to the values before the operation. This difference also persisted, although to a lesser extent, for the EI_max_ parameter (Figure 1B) characterizing the maximum of the EI-SS curve (*p* = 0.001 vs. before operation). After the operation, only the portal sample showed a significant difference (*p* = 0.045 vs. control portal vein). SS_1/2_ parameters (Figure 1C) deteriorated in the anastomosis group after the operation in the case of the systemic and portal sampling point (*p* = 0.008 vs. femoral artery, *p* < 0.001 vs. femoral vein, *p* = 0.003 vs. portal vein) but the difference between groups did not show a significant difference. The ratio (Figure 1D) slightly increased after the operation, mainly in the portal and renal sample (*p* = 0.001 vs. before operation), with a slight worsening in the systemic samples.

### 3.3. Osmotic Gradient Ektacytometry (Osmoscan)

**Figure 2:**
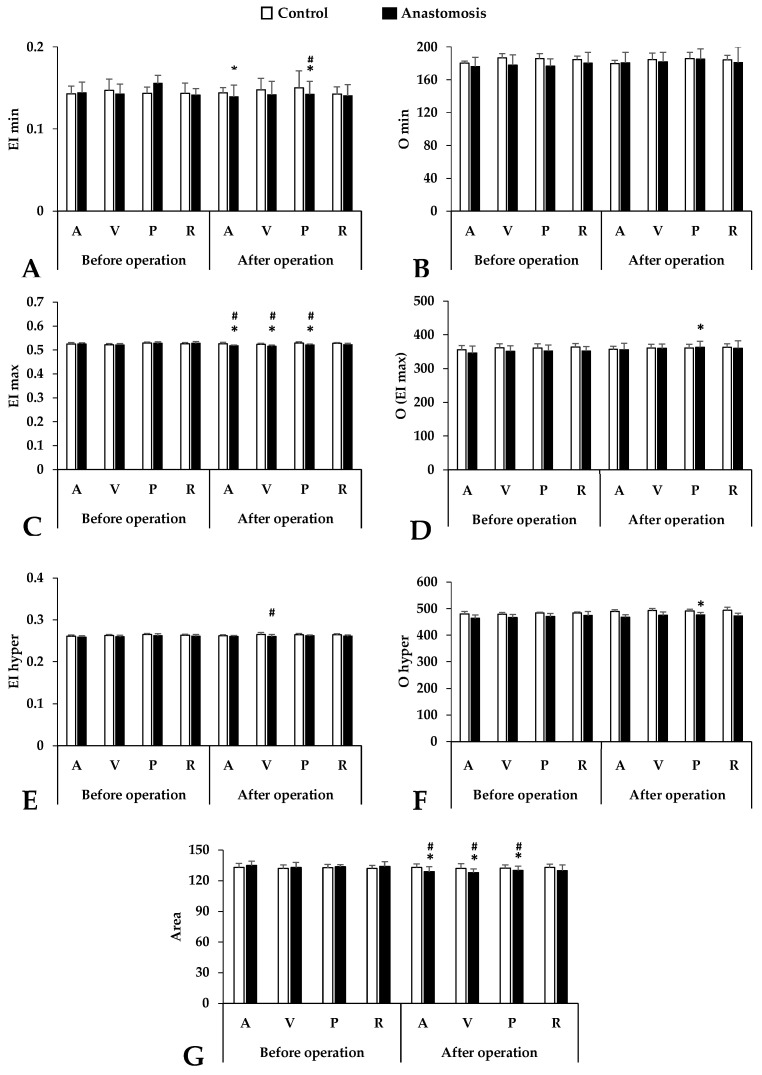
Osmotic gradient ektacytometry parameters in systemic arterial (A), venous (V), portal venous (P) and renal venous (R) blood taken before and after operation in the control and anastomosis groups. (**A**): EI min; (**B**): O min; (**C**): EI max; (**D**): O (EI max); (**E**): EI hyper; (**F**): O hyper; (**G**): Area under curve. Means ± S.D.; * *p* < 0.05 vs. before operation (base), # *p* < 0.05 vs. control.

Concerning osmotic gradient deformability values, the EI min parameter showed a slight decrease in all blood sampling points but the most noticeable difference was seen in the case of the portal vein sample after the operation (*p* < 0.001 vs. control portal vein, *p* = 0.001 vs. before operation) (Figure 2). 

This downward trend in the EI max continues. The most significant differences were observed in the femoral artery and vein and the portal vein (*p* < 0.001 vs. before operation, *p* = 0.001 vs. control femoral artery, *p* = 0.002 vs. control femoral vein, *p* < 0.001 vs. control portal vein). In the hyperosmolar environment, the red blood cell deformability also deteriorated in the anastomosis group but only the sample from the femoral vein showed a significant difference compared to the control group (*p* = 0.033 vs. control femoral vein). Osmolality parameters (O min, O (EI max), O hyper) also showed colorful diversity. We found a pattern similar to the elongation results. The most noticeable changes could be observed in the case of the O (EI max) and O hyper results, mainly in the portal vein samples after the operation. The area under the curve is a good description of the size and shape of the osmoscan curves. The smaller this value, the worse the deformability. Significant worsening was observable after surgery at three blood sampling points (femoral artery and vein, portal vein), and this difference is visible when these results are compared to those of the control group.

Table 2 provides a summary of other comparison metrics (ΔEI, ΔO, EI max/EI min, O (EI max)/O min, and their ratios). These extra parameters concentrate on the left region of the EI-O curves and represent how much EI varies as a function of osmolality in the hypo-osmolar direction (Table 2).

### 3.4. Red Blood Cell Aggregation

In the light-transmission-based aggregation measurement, significant differences in the aggregation index values were measured in stasis at the 5th second (M 5 s), 10th second (M 10 s), and at low shear rate (M1 5 and 10 s) after disaggregation was observed between the groups. 

**Figure 3:**
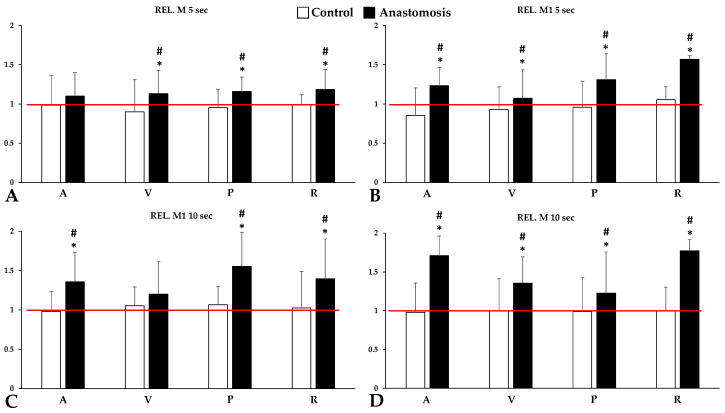
Relative values (versus base) of red blood cell aggregation parameters of systemic arterial (A), venous (V), portal venous (P) and renal venous (R) blood, tested for 5 or 10 s at stasis (M values, shear rate: 0 s^−1^) (**A**,**C**) or at a low shear (M1 values, shear rate: 3 s^−1^) (**B**,**D**). The red line represents the base (as 100%). Means ± S.D.; * *p* < 0.05 vs. before operation (base), # *p* < 0.05 vs. control.

Interestingly, this increase in red blood cell aggregation levels was only observed in the anastomosed group. In the control group, red blood cell aggregation showed only minimal variation in all parameters tested, which could be either non-significantly decreasing or increasing. The most marked increases in aggregation were seen for M1 5 s and M 10 s parameters in renal venous blood samples (Rel. M1 5 s: *p* < 0.001 vs. before operation and control; Rel M10 s: *p* < 0.001 vs. before operation and control). Notably, the portal and renal blood samples were significantly higher than the baseline and the control group in all aggregation parameters (Figure 3).

### 3.5. Blood Gas, Acid-Base and Metabolites

The oxygen partial pressure levels decreased after the bowel surgery in all cases in the anastomosis group, mainly in the femoral artery and portal sampling site (Femoral artery: *p* < 0.001 vs. before operation, *p* = 0.005 vs. control; portal vein: *p* < 0.001 vs. before operation, *p* < 0.001 vs. control). On the other hand, parallel with the oxygen pressure, the carbon dioxide levels increased after operation compared to the base values and the control results (Femoral vein: *p* < 0.001 vs. before operation, *p* = 0.005 vs. control; Portal vein: *p* < 0.001 vs. before operation, *p* < 0.001 vs. control; Renal vein: *p* < 0.001 vs. before operation, *p* < 0.001 vs. control). The pH results did not show much variation after blood sampling, as only a small change was seen in the femoral vein. Bicarbonate levels were significantly decreased in the portal and renal samples after the operation (*p* < 0.001 vs. before operation) but no significant difference was seen compared to the control group (Table 3).

Sodium and potassium levels also changed during intestinal surgery (Table 4). In contrast with the bicarbonate level, sodium and potassium levels showed a large difference compared to the control group. Calcium and chloride ion concentrations did not change significantly compared to the control group or the base measurements. 

As expected, glucose levels would increase slightly during surgery (both in the control and anastomosed groups) in the portal and renal samples of the anastomosis group, and this increase was significant (*p* = 0.045 vs. before operation). Examining lactate levels, we found that the effect of surgery showed a significant increase, and this difference was also significantly different compared to the control group (Femoral artery: *p* = 0.011 vs. before operation, *p* = 0.002 vs. control; Femoral vein: *p* < 0.001 vs. before operation, *p* < 0.001 vs. control; Portal vein: *p* < 0.001 vs. before operation; Renal vein: *p* < 0.001 vs. before operation, *p* < 0.001 vs. control). A small, non-significant increase in creatinine levels was observed during this study (Table 5).

**Figure 4:**
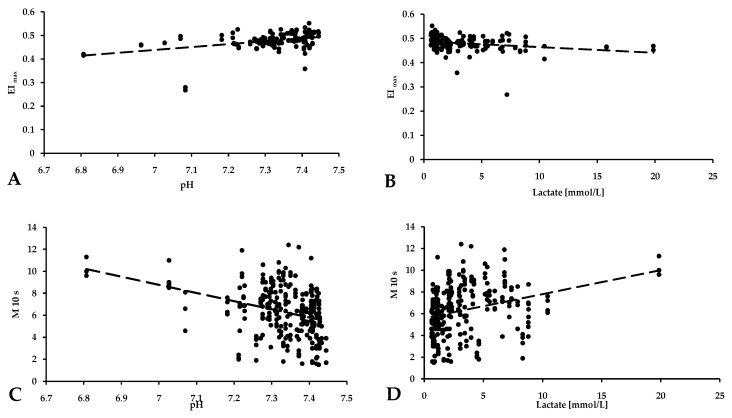
Correlation of the red blood cell maximal elongation index (deformability) (**A**,**B**) and aggregation values (**C**,**D**) with pH and lactate concentration.

We also observed that changes in pH and lactate concentrations, as described, correlated with changes in red blood cell deformability and aggregation. As the pH increased, and reached a physiological limit, the EI_max_ value increased in parallel (*p* = 0.003, R^2^ = 0.151) (Figure 4A). As the lactate concentration increased, the deformability of red blood cells deteriorated (*p* < 0.001, R^2^ = 0.773) (Figure 4B). Red blood cell aggregation indexes are also correlated with pH and lactate values. In general, red blood cell aggregation values increased with increasing lactate concentration (*p* < 0.001, R^2^ = 0.823) (Figure 4D) and decreasing pH (*p* = 0.002, R^2^ = 0.343) (Figure 4C).

## 4. Discussion

While the use of hemorheological investigations in research is growing, little is known about the in vivo importance of the variations in pathological processes that have been observed, including the effects of time, local-systemic rheology, and surgical procedures. The cumulative impact of these therapies is yet unclear, and a comprehensive knowledge of their combined hemorheological and microcirculatory effects is still challenging.

In our current study, we investigated how abdominal operation can affect micro- and micro-rheological parameters, and whether these parameters differ when samples are taken from different blood sampling points at different stages of the surgery. Over the past few decades, the utilization of modern hemorheological devices has led to a substantial increase in available data concerning regional hemorheological properties [21,22,23]. Additionally, it has provided insights into the comparison between local and systemic alterations in red blood cell deformability and aggregation within diverse pathophysiological conditions. These conditions encompass circulatory disorders, ischemia-reperfusion, and vascular anastomosis [21,29,30,31]. The use of minimal sample volumes and advanced methodologies has opened possibilities for in-depth research on micro-rheological variations between arteries and veins in laboratory animals. Furthermore, it addresses concerns regarding standardization when comparing systemic venous and capillary blood samples in humans [23,32]. The standardization of blood sampling techniques and sites is equally crucial for experimental surgical and microsurgical research models. Before blood collection, we should consider the species, genetic background, weight, sex, age, how often and how much blood we want to take, and which blood collection points to use. The amount of blood circulating is species- and body-weight-dependent. Pigs have 56–69 mL of blood per kilogram of body weight, depending on the breed. Generally, 10% of the circulating blood volume can be withdrawn without complications [33]. By adhering to these criteria, the parameters we study provide a reliable description of the impact of the intervention: the animals used in our research had about 1100 mL of circulating blood, of which only about 28 mL was taken during this study.

Investigating the hematological parameters, we found that the total leukocyte count was lower after surgery, while the red blood cell count, the mean corpuscular volume and the hematocrit increased. The most significant changes were found at portal and renal sampling sites. In our study, we discovered a pattern similar to previous research, with a lower white blood cell (WBC) count observed in arterial blood. It can be assumed that this difference may be due to the relative distributional variances in leukocytes or to the order of blood sampling, with the final sampling site being the abdominal aorta. However, we also observed higher red blood cell counts (calculated using the microcell counter from measured hematocrit and mean corpuscular volume values), hematocrit levels, and platelet counts in the systemic venous and portal venous blood samples [34,35]. Furthermore, the osmoscan enables the exploration of correlations between RBC deformability and key parameters such as mean corpuscular volume (MCV) and mean corpuscular hemoglobin concentration (MCHC). Altogether, the osmoscan emerges as a valuable instrument for comprehensively studying RBC deformability and its associations with physiological and pathological states [21].

Regarding the data on red blood cell deformability and osmotic gradient deformability, clear distinctions were observed among arterial, systemic, and portal venous blood in this experiment. Remarkably, arterial blood displayed the lowest elongation index values, while systemic venous blood exhibited the highest. The portal venous blood, on the other hand, showed EI values in between. Moreover, we saw significantly larger reductions in the operated group after the anastomosis compared to the control group. The largest changes were observed in the arterial and portal samples. Red blood cell deformability plays a crucial role in maintaining proper blood flow through the microvasculature, ensuring efficient oxygen delivery to tissues. However, after abdominal surgery, several interconnected factors can contribute to a decrease in RBC deformability, potentially impacting overall tissue oxygenation and patient recovery [36]. Inflammation is a key part of the healing process, but it can affect RBCs’ properties, leading to reduced deformability. This alteration can impede RBCs’ ability to pass through narrow capillaries and obstructed microvessels, affecting blood flow to vital organs [37,38]. Moreover, surgeries often involve substantial blood loss, which can lead to changes in blood composition. Reduced RBC volume and the introduction of older or damaged RBCs during blood transfusions can further contribute to decreased deformability [39]. Hemodilution, because of the administration of large volumes of intravenous fluids during surgery to maintain blood pressure and fluid balance, can also impact RBC deformability. The dilution of RBC concentration in the bloodstream affects their ability to function optimally [40,41]. Additionally, surgical procedures can subject the body to mechanical stress and trauma. This mechanical strain can directly damage RBCs or alter their behavior in circulation, reducing their deformability. Furthermore, the duration of surgery may lead to temporary episodes of reduced blood flow and oxygen delivery to tissues, causing hypoxia. Hypoxic conditions can impair RBC function and exacerbate the decrease in deformability [42]. Certain anesthetic agents and drugs used during surgery can also influence RBC deformability either directly or indirectly, further contributing to the observed decrease.

The data on red blood cell aggregation revealed pronounced differences. However, the four variables (M and M1 index values at 5 s and 10 s) did not exhibit similar distinctions. Nevertheless, in all cases, the aggregation index values were significantly higher after completing the anastomosis compared to the control group. The most notable degree of variation was observed in portal and renal samples. The search results currently available lack a definitive explanation for the immediate increase in red blood cell aggregation after surgery. Nonetheless, several potential factors may contribute to this phenomenon, including an inflammatory response, hemodilution, blood loss, and changes in shear stress or turbulence within the vessel system [43,44]. It is crucial to acknowledge that the reasons behind the heightened RBC aggregation post-operatively are likely to be multifactorial and can differ according to the patient’s characteristics and the specific surgical procedure performed.

To explore local versus systemic changes in red blood cell deformability and aggregation, factors such as cell oxygenation, blood pH, and lactate concentration are crucial [45,46]. Cicha and colleagues found that oxygenated red blood cells exhibited higher aggregation compared to deoxygenated cells, with the level of aggregation varying based on the method of deoxygenation [47]. Moreover, the rouleaux formation rate was positively correlated with increasing pH, with the lowest rates observed in oxygen/nitrogen and nitrogen/carbon dioxide incubations and the highest in air and nitrogen incubations. Uyuklu and colleagues examined the effect of oxygenation or deoxygenation on the aggregation and deformability of red blood cells and found that oxygenated samples had lower aggregation and better deformability compared to deoxygenated samples, and native control blood samples fell between these values [48]. These findings are essential for refining laboratory measurement techniques and standardizing sampling and handling conditions. As expected from physiological considerations, the arterial blood samples exhibited the highest pO_2_ values, while lower values were observed in renal and systemic venous blood samples. The lowest pCO_2_ values were recorded in arterial blood, with the highest values found in venous blood, and the portal blood samples fell in between. Blood pH showed similarity between systemic and portal venous blood; however, both glucose and lactate concentrations were lower in systemic venous and arterial blood samples. The investigation into the red blood cell aggregation index has yielded intriguing correlations with pH and lactate concentration values. These associations illuminate the complex relationship between hemorheological factors and metabolic status. The results indicate that changes in blood acidity and lactate levels might impact the tendency of red blood cells to aggregate, potentially influencing blood flow dynamics and tissue oxygenation. Additionally, investigations into parameters describing red blood cell deformability also show links with pH and lactate concentrations, suggesting a broader connection between the body’s physiological state and the mechanical properties of red blood cells [47,49].

This study’s limitations include the relatively small number of cases and the possibility of inter-species variances for extrapolation. We used a conventional bowel surgical procedure in this study; so, it may be worthwhile to investigate the effect of other surgical procedures with a similar experimental design. Microcirculatory changes are likely to underlie the micro-rheological variations we investigated, and understanding these may be aided by using additional measurement methods and a longer study period, including a follow-up period of several days. Supposedly, various co-morbidities may deepen further these findings, as hemorheological factors can be influenced by cardiovascular and metabolic disorders, resulting in impaired red blood cell deformability an enhanced erythrocyte aggregation. The number of red blood cells also influence the aggregation process, e.g., in various anemias or polycythemia [50,51].

## 5. Conclusions

Red blood cell deformability decreased significantly in both arterial and portal samples after surgery. Deformability was linked to pH and lactate levels: the RBC elongation index increased with higher pH, while a higher lactate concentration resulted in decreased deformability. Aggregation index values were highest in arterial blood and lowest in renal venous blood, showing a slight decrease by the end of the operation. Aggregation values correlated with pH and lactate, increasing with higher lactate and lower pH. 

In summary, micro-rheological parameters showed basic arterio-venous and porto-renal venous differences, influenced primarily by oxygenation level, pH and lactate concentration. The intestinal anastomosis operation caused immediate micro-rheological deteriorations with portal venous dominancy in this experiment. To better understand the significance and causes of these differences, additional comparative studies are needed.

## Figures and Tables

**Table 1:** Hematological parameters in systemic arterial (A), venous (V), portal venous (P) and renal venous (R) blood taken before and after operation in the control and anastomosis groups.

Variable	Group	Before Operation				After Operation			
		A	V	P	R	A	V	P	R
WBC [10^9^/L]	Control	15.86 ± 4.06	15.52 ± 2.89	14.78 ± 6.18	12.51 ± 4.52	15.89 ± 3.12	15.33 ± 3.02	15.19 ± 4.28	13.5 ± 4.5
	Anastomosis	19.19 ± 7.25	18.6 ± 7.6	19.11 ± 8.08	19.22 ± 6.65	13.24 ± 2.47 *#	12.74 ± 2.49 *#	14.12 ± 3.85 *	14.28 ± 2.96 *
RBC [10^12^/L]	Control	5.79 ± 0.44	5.81 ± 0.7	6.55 ± 1.1	6.37 ± 0.83	6.17 ± 0.71	6.32 ± 0.86 *	6.63 ± 0.48	6.52 ± 0.74
	Anastomosis	6.7 ± 0.55	6.93 ± 0.62	7.23 ± 0.55	6.85 ± 0.43	6.89 ± 0.34 #	7.37 ± 1.27 *#	8.05 ± 1.32 *#	7.37 ± 1.52 *#
Hgb [g/L]	Control	108.2 ± 11.3	111.8 ± 10.9	116.2 ± 10.2	111.1 ± 8.1	111.6 ± 7.2	112.8 ± 7.7	129.8 ± 20.1 *	117.9 ± 23.9
	Anastomosis	103.9 ± 9.2	103.9 ± 8.5	107.8 ± 33.9	113.8 ± 13.5	120.4 ± 26.5 *#	125.6 ± 24.8 *#	118.3 ± 16.6 *	116.6 ± 16.7
Hct [%]	Control	33.28 ± 2.78	33.48 ± 2.5	39.59 ± 6.27	36.98 ± 4.55	34.39 ± 3.87	34.02 ± 3.32	39.04 ± 3.37	36.89 ± 3.4
	Anastomosis	36.08 ± 3.77	37.56 ± 3.93	39.34 ± 3.5	37.17 ± 2.54	37.65 ± 2.34	38.29 ± 2.52	46.93 ± 6.63 *#	45.37 ± 7.8 *#
MCV [fL]	Control	57.47 ± 2.35	57.61 ± 2.35	58.52 ± 2.41	57.66 ± 2.07	57.8 ± 2.81	57.85 ± 2.75	58.04 ± 2.77	57.72 ± 2.7
	Anastomosis	53.75 ± 1.78	54.14 ± 1.67	54.37 ± 1.47	54.26 ± 1.39	55.53 ± 1.48 *#	55.88 ± 1.63 *#	56.02 ± 1.59 *#	56.39 ± 1.34 *#
MCH [pg]	Control	17.94 ± 0.88	17.91 ± 0.98	17.89 ± 0.97	17.94 ± 1.09	17.99 ± 0.83	18.08 ± 0.9	17.85 ± 0.83	17.84 ± 0.97
	Anastomosis	16.11 ± 0.6	16.13 ± 0.59	16.06 ± 0.6	16.21 ± 0.57	16.14 ± 0.63	16.14 ± 0.54	16.15 ± 0.6	16.01 ± 0.37
MCHC [g/L]	Control	312.13 ± 6.23	310.31 ± 6.58	305.94 ± 8.59	308.56 ± 9.49	310.81 ± 3.37	310.31 ± 6.44	305.56 ± 4.4	308.63 ± 5.63
	Anastomosis	300 ± 4.7	297.8 ± 7.1	295.6 ± 6.9	298.7 ± 4.7	300 ± 2.4 #	293.4 ± 8.4 #	291.4 ± 8.3 #	292.4 ± 6.5 #
Plt [10^9^/L]	Control	465.4 ± 107.9	472.6 ± 129.4	456 ± 132	423.6 ± 137.7	393.3 ± 123.1 *	376.1 ± 141.8 *	305.6 ± 95.8 *	418.8 ± 121.3
	Anastomosis	504.6 ± 107.7	464.1 ± 141.9	478.6 ± 139.6	478.8 ± 124.6	393.7 ± 126.3 *	409.9 ± 136.1 *#	393.2 ± 161.6 *#	396.67 ± 155.1 *

WBC: white blood cell count; RBC: red blood cell count; Hgb: hemoglobin; Hct: hematocrit; MCV: mean corpuscular volume; MCH: mean corpuscular hemoglobin; MCHC: mean corpuscular hemoglobin concentration; Plt: platelet count. Means ± S.D.; * *p* < 0.05 vs. before operation (base), # *p* < 0.05 vs. control.

**Table 2:** Additional comparative parameters of elongation index (EI)–osmolality (O) curves in systemic arterial (A), venous (V), portal venous (P) and renal venous (R) blood taken before and after operation in the control and anastomosis groups.

Variable	Group	Before Operation				After Operation			
		A	V	P	R	A	V	P	R
ΔEI	Control	0.383 ± 0.01	0.375 ± 0.02	0.385 ± 0.01	0.383 ± 0.02	0.382 ± 0.01	0.375 ± 0.01	0.379 ± 0.02 *	0.385 ± 0.01
	Anast.	0.381 ± 0.01	0.379 ± 0.01	0.372 ± 0.04	0.386 ± 0.01	0.379 ± 0.01	0.374 ± 0.02 *	0.378 ± 0.02 *	0.382 ± 0.01 *
ΔO	Control	175.63 ± 13.5	175.75 ± 8.2	175 ± 8.9	178.75 ± 9.6	176.88 ± 7.8	176.13 ± 5.1	175.25 ± 5	179.25 ± 6.9
	Anast.	169.57 ± 13.3	173.43 ± 9.1	175.43 ± 1.7	171.71 ± 7.1	175.71 ± 6.8 *	177.86 ± 10 *	178.14 ± 7.3	179.43 ± 13.3 *
ΔEI /ΔO	Control	0.002 ± 0.0001	0.002 ± 0.0001	0.002 ± 0.0001	0.002 ± 0.0001	0.002 ± 0.0001	0.002 ± 0.0001	0.002 ± 0.0001	0.002 ± 0.0001
	Anast.	0.002 ± 0.0001	0.002 ± 0.0001	0.002 ± 0.0001	0.002 ± 0.0001	0.002 ± 0.0001	0.002 ± 0.0001	0.002 ± 0.0001	0.002 ± 0.0001
rEI	Control	3.69 ± 0.21	3.59 ± 0.43	3.69 ± 0.19	3.7 ± 0.36	3.66 ± 0.07	3.56 ± 0.32	3.57 ± 0.42	3.72 ± 0.24
	Anast.	3.67 ± 0.32	3.67 ± 0.3	3.54 ± 0.68	3.74 ± 0.2	3.74 ± 0.26 *#	3.68 ± 0.47	3.7 ± 0.45 *#	3.75 ± 0.36
rO	Control	1.98 ± 0.08	1.94 ± 0.04	1.94 ± 0.04	1.97 ± 0.05	1.98 ± 0.05	1.96 ± 0.04	1.94 ± 0.04	1.97 ± 0.13
	Anast.	1.95 ± 0.07	1.98 ± 0.08	1.99 ± 0.12	1.96 ± 0.09	1.98 ± 0.05	1.98 ± 0.09	1.97 ± 0.06	2.01 ± 0.14
rEI /rO	Control	1.87 ± 0.12	1.85 ± 0.23	1.9 ± 0.1	1.88 ± 0.17	1.84 ± 0.06	1.82 ± 0.18	1.83 ± 0.21	1.88 ± 0.14
	Anast.	1.87 ± 0.16	1.86 ± 0.22	1.79 ± 0.41	1.92 ± 0.18	1.89 ± 0.15	1.87 ± 0.28	1.89 ± 0.27 *#	1.89 ± 0.29

ΔEI: the difference between maximal and minimal EI values; ΔO: the difference between osmolality values at maximal and minimal EI; rEI: ratio of maximal and minimal EI values (EI max/EI min); rO: ratio of osmolality values at maximal and minimal EI (O (EI max)/O min). Means ± S.D.; * *p* < 0.05 vs. before operation (base), # *p* < 0.05 vs. control.

**Table 3:** Partial pressure of oxygen and carbon dioxide, pH and bicarbonate concentration concentrations in systemic arterial (A), venous (V), portal venous (P) and renal venous (R) blood taken before and after operation in the control and anastomosis groups.

Variable	Group	Before Operation				After Operation			
		A	V	P	R	A	V	P	R
pO_2_ [mmHg]	Control	82.1 ± 8.7	45.4 ± 13.8	61.5 ± 19.5	50.7 ± 11.1	82.5 ± 6.5	45.1 ± 9.7	60.2 ± 7.9	55.6 ± 11
	Anastomosis	89.2 ± 1.6	49.9 ± 8.8	47.7 ± 11.7	44.9 ± 10.9	75.9 ± 5.5 *#	46.1 ± 7	36.6 ± 5.1 *#	42.1 ± 3.9
pCO_2_ [mmHg]	Control	48.3 ± 6.1	53.2 ± 4.1	62 ± 6.8	55.8 ± 8.3	49.1 ± 8.3	54 ± 6.9	61.8 ± 8.2	54.1 ± 9
	Anastomosis	53.3 ± 3	57.1 ± 3.7	65.5 ± 8.9	58.8 ± 9	54.1 ± 15.6	63.7 ± 2.6 *#	75.1 ± 7.6 *#	69.3 ± 7 *#
pH	Control	7.37 ± 0.02	7.33 ± 0.02	7.28 ± 0.04	7.33 ± 0.04	7.36 ± 0.05	7.33 ± 0.04	7.3 ± 0.06	7.35 ± 0.05
	Anastomosis	7.38 ± 0.05	7.36 ± 0.06	7.33 ± 0.05	7.38 ± 0.03	7.34 ± 0.01	7.25 ± 0.02 *#	7.25 ± 0.02	7.36 ± 0.01
cHCO_3_^−^ [mmol/L]	Control	27.94 ± 2.33	28.13 ± 1.93	29.26 ± 2.99	29.11 ± 2.64	27.29 ± 2.26	28.03 ± 1.88	28.96 ± 2.1	28.75 ± 2.04
	Anastomosis	31.31 ± 2.97	32.65 ± 3.33	36.2 ± 2.6	34.55 ± 3.12	28.84 ± 5.07	30.15 ± 5.16	31.12 ± 4.41 *	27.3 ± 6.72 *

Means ± S.D.; * *p* < 0.05 vs. before operation (base), # *p* < 0.05 vs. control.

**Table 4:** Sodium, potassium, calcium and chloride concentrations in systemic arterial (A), venous (V), portal venous (P) and renal venous (R) blood were taken before and after operation in the control and anastomosis groups.

Variable	Group	Before Operation				After Operation			
		A	V	P	R	A	V	P	R
Na^+^ [mmol/L]	Control	141.8 ± 2.2	142 ± 5.9	143 ± 2.6	142 ± 2.4	143.1 ± 3.2	143.1 ± 3.9	142.1 ± 3.4	142.6 ± 2.5
	Anastomosis	146.3 ± 4.2	149.5 ± 4.4	145.6 ± 1.9	148.6 ± 3.2	148.3 ± 1.2 *#	152.2 ± 2.2 #	149.8 ± 3.6 *#	149.1 ± 6.2 #
K^+^ [mmol/L]	Control	4.13 ± 0.6	4.29 ± 0.9	4.54 ± 0.5	4.01 ± 0.4	4.09 ± 0.3	4.24 ± 0.4	4.74 ± 0.2	4.1 ± 0.3
	Anastomosis	3.65 ± 0.5	3.66 ± 0.5	4.83 ± 0.9	4.38 ± 09	3.67 ± 0.7	3.62 ± 0.6	3.96 ± 0.5 *#	3.74 ± 0.5 #
Ca^2+^ [mmol/L]	Control	1.42 ± 0.1	1.44 ± 0.1	1.43 ± 0.1	1.43 ± 0.1	1.41 ± 0.1	1.43 ± 0.1	1.43 ± 0.3	1.43 ± 0.2
	Anastomosis	1.41 ± 0.1	1.4 ± 0.1	1.37 ± 0.1	1.38 ± 0.2	1.38 ± 0.1	1.38 ± 0.1	1.37 ± 0.2	1.38 ± 0.3
Cl^−^ [mmol/L]	Control	100.75 ± 3.2	100.38 ± 4.1	100.5 ± 1.2	100.25 ± 2	101.75 ± 1.8	101.25 ± 2.3	101.75 ± 1.5	100.75 ± 1.8
	Anastomosis	99.4 ± 3.3	98 ± 3.6	99.8 ± 4	98.8 ± 3.9	101.78 ± 6.3	101.2 ± 5.5	101.6 ± 4.3	101.88 ± 4.5

Means ± S.D.; * *p* < 0.05 vs. before operation (base), # *p* < 0.05 vs. control.

**Table 5:** Glucose, lactate and creatinine concentrations in systemic arterial (A), venous (V), portal venous (P) and renal venous (R) blood taken before and after operation in the control and anastomosis groups.

Variable	Group	Before Operation				After Operation			
		A	V	P	R	A	V	P	R
Glu [mmol/L]	Control	7.3 ± 2.3	6.9 ± 2.5	9 ± 2.4	7.7 ± 2.4	7.5 ± 2.2	7.1 ± 1.6	9.4 ± 2.4	8 ± 2.6
	Anastomosis	6.1 ± 3.4	6.2 ± 3.2	5.8 ± 3.3	6.1 ± 3.1	6.5 ± 3.5	7 ± 3.4	8.7 ± 2.1 *	8.1 ± 2.8 *
Lac [mmol/L]	Control	0.96 ± 0.23	0.84 ± 0.19	1.83 ± 1.06	1.02 ± 0.52	1.03 ± 0.36	0.94 ± 0.2	1.71 ± 0.65	0.97 ± 0.48
	Anastomosis	1.13 ± 1	1.22 ± 1.15	0.87 ± 0.4	0.82 ± 0.41	1.72 ± 1.4 *#	2.05 ± 1.79 *#	2.16 ± 1.18 *	1.96 ± 1.32 *#
Crea [µmol/L]	Control	106.5 ± 13.8	102.9 ± 18.5	111.4 ± 12.7	91.8 ± 22.6	109.5 ± 14.8	102.6 ± 18.1	110.7 ± 17.1	89.9 ± 16.2
	Anastomosis	94.5 ± 10.1	95.3 ± 17.8	106.4 ± 25.2	86.5 ± 26.9	100.4 ± 20.1	103.8 ± 27.2	114.4 ± 29.5	88.8 ± 19

Means ± S.D.; * *p* < 0.05 vs. before operation (base), # *p* < 0.05 vs. control.

## Data Availability

The data presented in this study are available on request from the corresponding author. The data are not publicly available due to ethical permission constraints.
